# Direct identification of *ALK* and *ROS1* fusions in non-small cell lung cancer from hematoxylin and eosin-stained slides using deep learning algorithms

**DOI:** 10.1038/s41379-022-01141-4

**Published:** 2022-09-03

**Authors:** Chen Mayer, Efrat Ofek, Danielle Even Fridrich, Yossef Molchanov, Rinat Yacobi, Inbal Gazy, Ido Hayun, Jonathan Zalach, Nurit Paz-Yaacov, Iris Barshack

**Affiliations:** 1 Institute of Pathology, Sheba Medical Center, Ramat Gan, Israel; 2 Imagene AI, Tel Aviv, Israel; 3 Sackler faculty of Medicine, Tel Aviv University, Tel Aviv, Israel

## Abstract

Anaplastic lymphoma kinase (*ALK*) and ROS oncogene 1 (*ROS1*) gene fusions are well-established key players in non-small cell lung cancer (NSCLC). Although their frequency is relatively low, their detection is important for patient care and guides therapeutic decisions. The accepted methods used for their detection are immunohistochemistry (IHC) and fluorescence in situ hybridization (FISH) assay, as well as DNA and RNA-based sequencing methodologies. These assays are expensive, time-consuming, and require technical expertise and specialized equipment as well as biological specimens that are not always available. Here we present an alternative detection method using a computer vision deep learning approach. An advanced convolutional neural network (CNN) was used to generate classifier models to detect *ALK* and *ROS1*-fusions directly from scanned hematoxylin and eosin (H&E) whole slide images prepared from NSCLC tumors of patients. A two-step training approach was applied, with an initial unsupervised training step performed on a pan-cancer sample cohort followed by a semi-supervised fine-tuning step, which supported the development of a classifier with performances equal to those accepted for diagnostic tests. Validation of the *ALK/ROS1* classifier on a cohort of 72 lung cancer cases who underwent *ALK* and *ROS1*-fusion testing at the pathology department at Sheba Medical Center displayed sensitivities of 100% for both genes (six *ALK*-positive and two *ROS1*-positive cases) and specificities of 100% and 98.6% respectively for *ALK* and *ROS1*, with only one false-positive result for *ROS1*-alteration. These results demonstrate the potential advantages that machine learning solutions may have in the molecular pathology domain, by allowing fast, standardized, accurate, and robust biomarker detection overcoming many limitations encountered when using current techniques. The integration of such novel solutions into the routine pathology workflow can support and improve the current clinical pipeline.

## Introduction

Lung cancer is the leading cause of cancer-related mortality and accounts for ~1.76 million deaths per year worldwide. Non-small cell lung cancer (NSCLC) comprises ~85% of all lung cancers and typically presents at advanced stages. The introduction of genotype-directed targeted therapies for NSCLC has transformed the care of patients and dramatically improved survival^1^.

Several tyrosine kinase inhibitor (TKI) targeted therapies are currently used for the treatment of lung cancer. The current College of American Pathologist (CAP)/International Association for the Study of Lung Cancer (IASLC)/Association for Molecular Pathology (AMP) guidelines recommend screening advanced-stage lung cancer patients for targetable alterations including testing for *EGFR*, *ALK*, and *ROS1*^2^. The National Cancer Center Network (NCCN) guidelines go even further and recommend broad molecular profiling using multiplex mutation screening assays or next-generation sequencing (NGS)^3^.

Despite rapid progress in the field, many practical issues often prevent or delay the initiation of targeted therapies. These obstacles include insufficient tissue for testing, poor quality of DNA or RNA, sequencing failure (3–10%)^4,5^, high cost, and high turnaround times^6^. Effective approaches to reducing these barriers are vitally needed to ensure patients’ access to available treatments.

Anaplastic lymphoma kinase (*ALK*) belongs to the insulin-like tyrosine kinase (TK) receptor superfamily. Its rearrangements occur in ~5% of NSCLC cases and are associated with younger age, no or light smoking history, and characteristic histology such as signet-ring cells and cribriform pattern^7^. *ROS* proto-oncogene 1 (*ROS1)* gene belongs to the subfamily of TK insulin receptor genes. Its rearrangements occur in ~1–2% of patients with NSCLC and are also commonly found in younger patients, no or light smokers, and are associated with histological features of adenocarcinoma and similar characteristics to *ALK-fusions* such as signet-ring and cribriform pattern^8,9^. While there are features associated with these alterations, none are in high enough association nor specific enough to be used as markers for fusions. Thus, all NSCLC patients are currently tested using conventional laboratory tests such as IHC, FISH and NGS as discussed in the manuscript. Both *ALK* and *ROS1* rearrangements result in fusion proteins with constitutive TK activity. A variety of genes have been identified as their fusion partners, most frequently *EML4* for *ALK* and *CD74 for ROS1*^10^. *ALK* fusions can effectively be blocked by TKIs, including alectinib, lorlatinib, brigatinib, crizotinib, and ceritinib^3^. Since *ALK* and *ROS1* are closely related kinases, many of these TKIs are also used for the treatment of *ROS1*-positive NSCLC patients^11^.

The initial gold standard method for detecting *ALK* and *ROS1* rearrangements was fluorescence in situ hybridization (FISH). Immunohistochemistry (IHC) was later added as an accepted alternative to FISH for *ALK* and as a screening method for *ROS1*, requiring subsequent confirmation of positive cases by another molecular or cytogenetic method^12,13^. Additional methods for identification of *ALK* and *ROS1-*rearrangements include reverse transcription PCR (RT-PCR) and NGS^14^. Each of these methods has its advantages and disadvantages, and the concordance between them varies between 70.7–98% depending on the gene and the assays used^2,15^. FISH, while still the gold standard, is expensive, time-consuming, and requires technical expertise and specialized equipment. Moreover, both false-negative and -positive cases of *ALK* and *ROS1-* rearrangements have been documented^16,17^. The use of IHC to detect *ALK* rearrangements has reported sensitivities and specificities ranging from 95% to 100%^2^. However, several studies have described discrepancies between FISH and IHC methods, with <90% concordance for *ALK* and only 80% concordance reported for *ROS1* testing^15,18^. The challenges facing both NGS and RT-PCR methodologies include a high technical failure rate (as much as 10–30% for NGS)^19^, partly due to the poor DNA/RNA quality in some formalin-fixed paraffin-embedded (FFPE) samples, as well as other limitations such as tissue scarcity and high cost, which hinder the widespread clinical implementation of these methods.

Deep learning (DL) is a subset of Artificial intelligence (AI) that uses machine learning (ML) algorithms in artificial neural networks to detect complicated patterns in large datasets. Computer vision is a field of AI that trains computers to interpret and understand the visual world using digital images. DL has revolutionized computer vision and has become a standard technique for image classification and is increasingly used to improve clinical practice across the medical imaging domain in a wide range of applications such as CT, MRI and mammography^20^. The advances in digitalization of tissue slides, alongside the evolving utilization of DL in the biomedical field, together with the increasing demand for molecular profiling in cancer, have led to the recognition of the potential for DL use in cancer classification and prediction of biomarker status. Oncogenic driver mutations are most probably reflected in an altered morphological pattern of the cancer cells and/or tissue. While not visible to the human eye, these changes can be detected by vision-based algorithms and are distinctive to each specific alteration. Indeed, there is an increasing number of publications describing the application of DL in the detection of molecular changes in histological slide images. Examples include, among others, prediction of PD-L1 (*n* = 82 test cases)^21^, *EGFR, KRAS*, and *TP53* (*n* = 59 test cases)^22^ alterations in NSCLC, MSI in colorectal cancer (*n* = 479 test cases)^23^, and ER/PR/HER2 status in breast cancer (*n* = 2611–2714 test cases)^24^. While these studies demonstrate the potential of AI in the molecular diagnosis of cancer, the results are not yet robust enough for the technique to replace the currently used genetic and molecular tests. To prove the robustness of the method, further studies should be performed on larger and more diverse clinical cohorts in order to demonstrate comparable performance to the standard of care.

Here we present the use of an AI-based solution to detect genomic rearrangements in *ALK* and *ROS1* from automatically scanned hematoxylin and eosin (H&E) stained lung tumor slides. Our work highlights the advantages of this AI solution, which provides a rapid, cost-effective, and accurate testing alternative. Integration of such solutions within the clinical practice can support pathologist workflow and assist oncologists to tailor the best treatment for optimal management of patient care, bridging the current gap towards true implementation of precision medicine.

## Materials and methods

### Sample collection

Digitized images of H&E slides from advanced-stage NSCLC patients were collected from the Pathology Department of Sheba Medical Center (according to IRB 7451-20-SMC). All images were scanned at 40× magnification using the Philips IntelliSite Ultra-Fast scanner (Philips Digital Pathology Solutions, Best, Netherlands) and converted into TIFF format.

### Data

A total of 234 NSCLC cases tested for *ALK* and *ROS1* rearrangements at Sheba Medical Center between 2012–2021 were selected for the study. These were randomly divided into training (*N* = 162) and validation (*N* = 72) sets. *ALK and ROS1* status was determined based on IHC, FISH, or NGS. Approximately 80% of patients in the validation set were tested by at least two testing methods (Supplementary Table S1). The training set included H&E scanned images as well as *ALK* and *ROS1* status annotations, while the validation set included images only.

### Fixation and H&E staining

Tissues were processed according to standard pathological procedures. Tissues were fixed in buffered formalin, embedded in paraffin, and sectioned using a microtome. Sections were then placed on histologic slides and stained using H&E.

### *ALK* (D5F3) and *ROS1* immunostaining

For the immunohistochemical studies, 4μm wide sections were prepared from FFPE blocks and positive control was added at the edge of the slides. IHC staining was performed using *ALK* (clone D5F3, V790-4794, Ventana Medical Systems Inc., Oro Valley, AZ, Oro Valley, AZ, USA) and *ROS1* (1:50; 3287S, Cell Signaling, Danvers, MA, USA) antibodies with the OptiView Amplification Kit (Ventana Medical Systems Inc.) in conjunction with the OptiView detection kit (Ventana Medical Systems Inc.) on a Benchmark Ultra staining module (Ventana Medical Systems Inc.). All cases were tested by a single and dedicated thoracic pathologist. As indicated by the International Association for the Study of Lung Cancer (IASLC) intensity scoring was as follows: strong staining (3+) is clearly visible using 2× or 4× objective lens, moderate staining (2+) requires a 10× or 20× objective lens and weak (1+) cannot be seen until a 40× objective is used. Cases with a diffuse staining and (3+) score were interpreted as positive.

### *ALK* and *ROS1* molecular analysis

For *ALK* and *ROS1* fusion analysis, DNA and RNA were extracted from paraffin-embedded sections using the KingFisher kit according to the manufacturer’s instructions. The concentration of extracted DNA and RNA was measured using NanoDrop 2000c Spectrophotometer (Thermo Fisher Scientific, Waltham, MA, USA) following DNA measurement using a Qubit Fluorometer (Thermo Fisher Scientific) and brought to a concentration of 25 ng/μl.

cDNA was synthesized from RNA using the Ion AmpliSeq™ HiFi Mix (Thermo Fisher Scientific). Sequencing was performed on the Ion Torrent System (Thermo Fisher Scientific).

### FISH analysis

*ALK* and *ROS1* rearrangement detection was performed using Vysis LSI *ALK* (2p23) Dual Color Break Apart Rearrangement FISH Probe (Abbott Molecular, Abbott Park, IL, USA) and Zytolight SPEC *ROS1* Dual color Breakpart Probe (ZytoVision GmbH, Bremerhaven, Germany). Assays were performed as described previously^25^.

### Algorithm development and validation

**Fig. 1:**
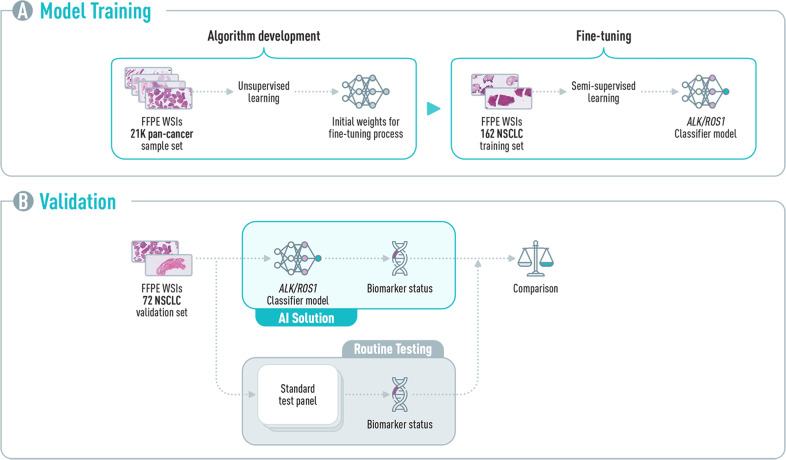
AI algorithm development and validation. **A** A two-step approach was used to generate an *ALK/ROS1* classifier; first, initial weights were generated from WSIs from Imagene-AI’s internal database, using an unsupervised learning algorithm. This was followed by a fine-tuning step performed using a semi-supervised algorithm, specifically for the tested biomarkers. **B** A retrospective set of de-identified scanned H&E images of 72 NSCLC samples were collected and analyzed by the *ALK/ROS1* classifier. Results of the AI solution and the routine practice (IHC, NGS, and/or FISH) were compared for performance assessment. FFPE Formalin-Fixed Paraffin-Embedded, WSI whole image slide.

To achieve high model accuracies, the development of the DL model was performed in two steps: (1) unsupervised learning training to produce initial weights for the neural network architecture; and (2) algorithm fine-tuning using semi-supervised learning. Following model generation, a retrospective blinded validation was performed (Fig. 1).

#### Unsupervised learning training

21,299 H&E-stained, FFPE pan-cancer, untagged whole slide images (WSI) were used from Imagene-AI LTD.’s internal database. All slides were de-identified and scanned at 40× magnification (0.23–0.27 μm per pixel resolution). No additional data was linked to the images. Data augmentation was applied to images using image pre-processing algorithms and generative adversarial networks (GANs). For this step, convolutional neural network (CNN) training was performed with an average of 87,000 image tiles (1536 × 1536 pixel) per WSI extracted using an on-the-fly image tile selection method (implemented to achieve random sampling of regions without the need for a priori creation of patches) fed through the network.

#### Algorithm fine-tuning

162 H&E WSI cases of NSCLC biopsies were retrieved from Sheba Medical Center’s archives and scanned at 40× magnification (0.25 μm per pixel resolution) using a Philips Ultra-Fast Scanner (Philips Digital Pathology Solutions). The training set included 15 *ALK*- and 7 *ROS1*-positive cases identified by gold standard testing (IHC, FISH and/or NGS). Data augmentation was applied on images using image pre-processing algorithms and GANs. To create the *ALK/ROS1* classifier algorithm, fine-tuning of the initial neural network algorithm developed in the previous step was conducted using semi-supervised learning and a five-fold cross-validation scheme. First, DL algorithms were trained to detect regions of interest (ROIs) in the WSIs (Supplementary Fig. S1). Next, model training was conducted on image tiles (384 × 384 pixels) extracted from the ROI. For image tile extraction an on-the-fly image tile sampling method was used, with an average of 1 M tiles per WSI generated and used for the training. In a few instances, when a couple of slides from the same cases were available, the first serial slide was used. Retrospectively, we tested all the consecutive slides, with 100% concordance.

#### Algorithm validation

For the evaluation of the *ALK/ROS1* classifier algorithm accuracy, NSCLC biopsies processed at Sheba Medical Center were used. A cohort of 72 de-identified WSI retrieved from Sheba Medical Center’s archives was used as a double-blinded set. The algorithm status predictions were compared to the ground truth diagnosis, and specificity and sensitivity were calculated to assess the AI-based model’s performance.

### Inference of molecular status

Images were uploaded into the AI solution powered by Imagene-AI LTD. in a local station as de-identified H&E scanned images, without additional clinical information.

A categorical prediction was made by the *ALK/ROS1* classifier model.

## Results

### *ALK* and *ROS1* detection by conventional methods

In this study, we compared the performance of *ALK* and *ROS1* conventional testing methods to that of the AI solution. IHC, FISH and NGS were used as gold standard methods for the analysis of these genes’ rearrangements. In total, 53 of the 72 cases in our cohort were subjected to NGS testing, 14 underwent FISH testing for *ALK*, and 7 for *ROS1* (Supplementary Table S1). *ALK* IHC was performed on 70 cases and *ROS1* IHC on 65 cases. Six out of 72 cases were detected as *ALK*-positive and two out of 68 cases as *ROS1*-positive. Five of the six *ALK*-positive cases were detected as positive by FISH, with one confirmed by NGS. The sixth case was identified by a combination of IHC and NGS. The two *ROS1*-positive cases were detected by FISH analysis.

### Validation of the *ALK*/*ROS1* classifier

The *ALK/ROS1* classifier algorithm was developed as described in the methods. Briefly, models were developed and fine-tuned on a training set which included H&E scanned images together with *ALK* and *ROS1* status annotations only. To validate the model, a retrospective cohort of 72 samples was tested with the AI solution using de-identified images data only (a representative heatmap of a patch for each alteration is presented in Supplementary Fig. S2). We then compared the results of the algorithm predictions with the results of the conventional methods described above. The model correctly inferred the status of all cases except for a single *ROS1* false positive. This sample was analyzed by all traditional methods (IHC, FISH, and NGS), which did not identify any *ROS1* alteration. Altogether, the model achieved a sensitivity of 100% for both genes and specificity of 100% and 98.57% for *ALK* and *ROS1* respectively (Table 1). The negative predictive value (NPV) for *ALK* and *ROS1* was 1, and the positive predictive value (PPV) was 1 for *ALK*-fusion and 0.505 for *ROS1*-fusion.

**Table 1:** Summary of *ALK/ROS1* classifier results.

		Conventional methods		AI-based model						
	N	# Positive	# Negative	TP	TN	FP	FN	Sensitivity	Specificity	Concordance
*ALK*	72	6	66	6	66	0	0	100%	100%	100%
*ROS1*	68	2	66	2	65	1	0	100%	98.48%	98.53%

*TP* true positive, *TN* true negative, *FP* false positive, *FN* false negative.

Traditionally, AI model performance is measured using AUC (Area Under the ROC Curve) values. However, here we chose to evaluate our model’s performance using the same characteristics as those employed to evaluate the assays used in clinical practice. The clinical performance of such assays (e.g., sequencing, IHC and FISH) is described by their sensitivity, specificity, and concordance with the current gold-standard tests. We thus reported the results accordingly; however, in accordance with the high performances, the AUC values were high as well, 1 for *ALK-*fusion and 0.986 for *ROS1*-fusion.

Of note, approximately 7% of samples analyzed by IHC (*N* = 9) were reported as “equivocal” and four samples tested by NGS failed (7.3%) due to technical issues such as poor DNA/RNA quality and insufficient diagnostic material. For diagnosis, these samples required additional testing with an alternative method. In contrast, all the tested samples in the cohort (including the aforementioned samples) were successfully analyzed by the AI pipeline.

### Method comparison

To examine the performance of the traditional methods and the AI solution, we compared the outcomes of the various methods. When comparing the conventional methods, two samples had conflicting results, negative by IHC and positive by FISH analysis. These cases were given an official result of *ALK*-positive according to the FISH analysis, which was in concordance with the *ALK/ROS1* classifier calling.

Comparison of the AI solution with the routine methods identified only a single case with conflicting results. In this case, IHC, FISH, and NGS analysis did not detect any rearrangement, while the *ALK/ROS1* classifier reported this case as *ROS1*-positive.

## Discussion

In this study, we used an AI-based solution to detect *ALK* and *ROS1* fusions in NSCLC cancer patients. To the best of our knowledge, this is the first study that has successfully applied an AI-solution on clinical samples to identify *ALK* and *ROS1* rearrangements directly from H&E pathological WSI of lung cancer patients. Despite the low frequency of alterations in these oncogenes (5 and 1–2% of NSCLC cases for *ALK* and *ROS1*, respectively), their detection is of high importance, as it determines clinical treatment^11^. Both IHC and FISH were initially used to detect these rearrangements. However, advances in technologies in parallel with the continued increase in the number of actionable biomarkers, have led to the incorporation of NGS panels in clinical diagnostics. Unfortunately, all these assays have limitations, and testing rates are variable in the clinical practice, with cost, quality standards, and access being substantial barriers^6,19^. Furthermore, the current ASCO/CAP/IASLC/AMP guidelines’ recommendations refer to a turnaround time of ten working days between sample receipt and reporting of the molecular test^26^, yet in practice most institutes have a much longer turnaround time of several weeks from the pathology review to the final molecular report^27^.

One of the major challenges in lung cancer screening is the limited amount of patient specimens. In 10–20% of cases tissue is insufficient for comprehensive sequencing or analysis by alternative methods^28^. This will most probably be less of a challenge to AI classifiers, as reflected in this study, where, unlike conventional methods, all the samples in the cohort received a biomarker status calling. The median number of patches per slide that went through the AI analysis was 266 (ranged: 4–7055) per case. The ability to infer biomarker status from as little as four patches per slide is of major importance in this context and implies that AI-based solutions may be less sensitive to limiting amounts of biopsied tissues.

Routine molecular testing in lung cancer presents several challenges, some of which we faced in our cohort. The first, is the commonly encountered technical failure of NGS reactions. In our validation set, 5.5% of samples that underwent sequencing failed. Secondly, IHC screening can sometimes have ambiguous results. Approximately 7% of our validation set was “equivocal” in the initial IHC screening, requiring additional tests. Thirdly, the use of multiple tests (FISH, NGS, and IHC) may sometimes result in conflicting interpretations. Indeed, relatively high levels of discordance between tests have been reported^2,15,18^. In our validation set, six of the eight positive cases were tested by at least two methods. Of these, two cases had conflicting results (negative in IHC and positive in the FISH assay). Two additional cases were equivocal in IHC and positive according to the FISH analysis (one for *ALK* and one for *ROS1*). The remaining two cases showed concordance between methods, representing only a third of the positive cases. This issue is of major importance, especially in positive cases, where identification of *ALK* or *ROS1* fusions dictates targeted treatment regimens.

Of the 140 tests performed using the AI solution (72 for *ALK* and 68 for *ROS1*), only a single case showed discordance with the final pathology report, demonstrating high accuracy of the AI solution. Further testing will be required to ascertain the robustness of this assay, and to see whether it will maintain its accuracy in real-world scenarios with larger datasets and when implemented in additional clinical settings and medical centers. Nonetheless, the diagnostic accuracy of the AI solution presented here, which was at least comparable if not even higher than that of the gold-standard tests, suggests that AI-based molecular diagnostic solutions may be appropriate as clinical diagnostic tools in the near future.

The technology used here examined the existence of *ALK* or *ROS1* fusions in NSCLC, irrespective of their partnering genes. This is in contrast to NGS for example, which can capture the identity of the fusion partner as well. Current targeted treatments for *ALK* and *ROS1* rearrangement disregard the partnering genes’ identity and there is limited information regarding any difference in response between them^7^. Thus, we generated a model that detects *ALK* and *ROS1* fusions, regardless of their precise partner. However, each partner can result in a fusion product with slightly different expression and/or activity. Such differences might be identifiable by AI models if deemed to be required, but this will necessitate a significantly larger dataset.

**Fig. 2:**
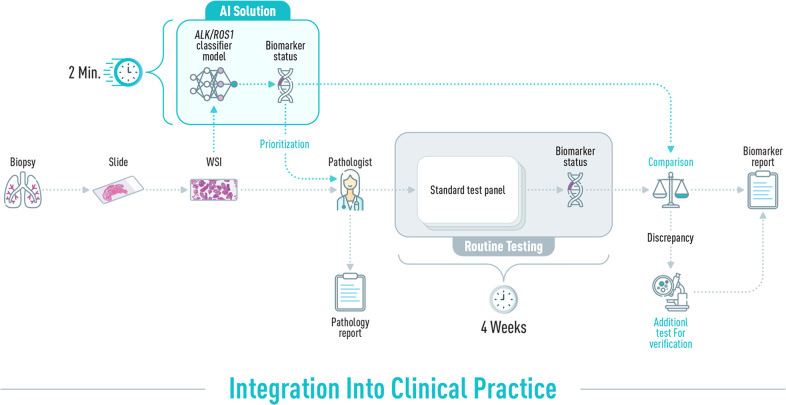
Integration of AI-based solutions in the clinical pipeline. Schematic overview of the integration of the AI-solution into the routine pathological biomarker diagnostic practice. After the biopsy is cut and stained with H&E, it is digitally scanned and transferred for pathological examination. Samples are further processed by the traditional route including IHC screening as well as FISH and NGS, to assess *ALK* and *ROS1* status. A final biomarker report is produced after an average of four weeks. The AI software (Imagene-AI. LTD) automatically analyses the WSI directly after the scanning of the H&E slide, taking an average of 2 min for *ALK/ROS1* status inference. Flagging of samples carrying *ALK* or *ROS1* alterations can be viewed immediately after this process by the pathologist and assist in their further analysis prioritization. After the results of the routine test panel are ready, they can be compared to the AI classifier inference. Discrepant cases can then be subjected to additional testing and clarification. AI artificial intelligence, WSI whole image slide, IHC Immunohistochemistry, FISH Fluorescence in situ hybridization, NGS Next-generation sequencing.

Integration of an AI solution within routine clinical pathological workflow holds great advantages. Figure 2 illustrates the integration of the AI solution directly after the scanning of the H&E slide enabling the biomarker status calling within minutes. The suggested workflow can expedite the time that elapses between biopsy and commencing targeted therapy by assisting pathologists in flagging samples that potentially carry alterations in real-time to prioritize their examination. Moreover, after completion of the conventional testing, samples that have discordance in their biomarker status between the routine tests and the AI solution can undergo additional confirmational tests before reporting back to the patient. Hence, the AI solution can improve biomarker detection accuracy and assist or even change patients’ testing prioritization.

Applying AI in clinical diagnosis can offer standardization and reproducibility, compared to current assays that suffer from subjectivity and wide variation between different labs and kits. Integration of AI medical software can also expand the availability of biomarker analysis in oncology as they do not require the expertise and specialized equipment that traditional assays demand, while preserving clinical standards of test accuracy. However, as opposed to molecular and biochemical assays that measure specific and known parameters, the outcome of the AI solution inference is based upon factors that are complex, obscure and undefinable. As technology progresses and the use in clinical practice is extended, new features will need to be developed to elucidate the morphological patterns used by the algorithm for analysis.

Advances in oncology and the gradual adoption of digitization of WSIs have paved the way for the incorporation of AI tools in digital pathology. This approach can improve our ability to tackle complex processes involved in cancer development. The concept of inferring molecular alterations directly from the morphometric phenotypes of tissue specimens presents a significant advantage compared to other analytical tools. This highlights a major advantage of AI-based methodology, such as the convolutional neural network (CNN) used here, which learns to identify patterns associated with the inferred alteration without the need for any prior annotations (such as histopathological features). By identifying different alterations that affect the cells and/or tissue morphology, DL algorithms can predict which tumors carry clinically significant changes without requiring a priori knowledge regarding the nature of the alteration (*i.e*., fusion partner, breakpoints, etc.). This can be applied to a wide range of biomarkers associated with different conditions such as mutations, protein expression, signatures, additional structural variants and more.

The molecular and IHC methods described in this study to diagnose *ALK* and *ROS1* alterations are used in most countries for advanced cases only. Among other reasons, cost and the overload of molecular exams since their introduction to oncology contribute to the limited number of patient samples ultimately sent for testing. Those eligible for molecular testing often wait several, excruciating weeks for results, time that can be crucial in advanced cancer stages. An AI-based solution such as that validated in this study, is a fast application, requiring no additional tissue, and can be widely implemented potentially for all newly diagnosed NSCLC patients regardless of stage. Furthermore, automatization of such analysis can reduce potential human and technical errors. Moreover, utilizing a single histological slide image for assessment of multiple biomarkers can align with the increasing decline in bio-specimen availability for molecular testing. Broad implementation can be relatively easily applied and while this may pose new clinical dilemmas, it should ultimately result in benefit for the patients.

## Supplementary information

Supplementary information

## Data Availability

The datasets generated during the current study are not publicly available and are available from the corresponding author, CM, upon reasonable request.
