# Supplementary material for: Direct identification of ALK and ROS1 fusions in non-small cell lung cancer from hematoxylin and eosin-stained slides using deep learning algorithms
Source: Mod Pathol. 2022 Sep 3;35(12):1882–7. doi: 10.1038/s41379-022-01141-4 (PMC9708557; doi:10.1038/s41379-022-01141-4)
Supplement: Supplementary file 1 — Supplementary information [file 41379_2022_1141_MOESM1_ESM.pdf]

Supplementary Information

Supplementary Table S1: Summary of testing of validation cohort samples

Case	ROS1 IHC	ROS1 NGS	ROS1 NGS	ROS1 FISH	Offitial result	ROS1 AI	ALK IHC	ALK NGS	ALK NGS	ALK FISH	Offitial result	ALK AI	concordance	Pathological type
1	Negative	RNA	Negative	Not performed	Negative	Negative	Negative	RNA	Negative	Not performed	Negative	Negative	YES	Lung adenocarcinoma metastasis to liver
2	Equivocal	RNA	Negative	Negative	Negative	Negative	Equivocal	RNA	Negative	Negative	Negative	Negative	YES	Lung Adenocarcinoma metastasis to Ilium
3	Negative	RNA	Negative	Not performed	Negative	Negative	Negative	RNA	Negative	Not performed	Negative	Negative	YES	NSCLC, Adenocarcinoma
4	Negative	RNA	Negative	Not performed	Negative	Negative	Negative	RNA	Negative	Not performed	Negative	Negative	YES	M/p lung adenosquamous carcinoma metastasis to liver
5	Negative	RNA	Negative	Not performed	Negative	Negative	Negative	RNA	Negative	Not performed	Negative	Negative	YES	NSCLC, Adenocarcinoma
6	Negative	RNA	Negative	Not performed	Negative	Negative	Negative	RNA	Negative	Not performed	Negative	Negative	YES	NSCLC, Squamous cell carcinoma
7	Negative	RNA	Negative	Not performed	Negative	Negative	Negative	RNA	Negative	Not performed	Negative	Negative	YES	Lung adenocarcinoma metastasis to Adrenal gland
8	Negative	RNA	Negative	Not performed	Negative	Negative	Negative	RNA	Negative	Not performed	Negative	Negative	YES	NSCLC, Adenocarcinoma
9	Not performed	RNA	Not performed	Not performed	not tested	Negative	Equivocal	RNA	Not performed	Negative	Negative	Negative	YES	NSCLC, Adenocarcinoma
10	Negative	RNA	Negative	Not performed	Negative	Negative	Negative	RNA	Negative	Not performed	Negative	Negative	YES	NSCLC, Adenocarcinoma
11	Negative	RNA	Negative	Not performed	Negative	Negative	Negative	RNA	Negative	Not performed	Negative	Negative	YES	NSCLC, sub-type not found
12	Negative	RNA	Negative	Not performed	Negative	Negative	Negative	RNA	Negative	Not performed	Negative	Negative	YES	NSCLC, sub-type not found
13	Negative	RNA	Negative	Not performed	Negative	Negative	Negative	RNA	Negative	Not performed	Negative	Negative	YES	Poorly differentiated carcinoma
14	Negative	RNA	Negative	Not performed	Negative	Negative	Negative	RNA	Negative	Not performed	Negative	Negative	YES	NSCLC, Adenocarcinoma
15	Negative	RNA	Negative	Not performed	Negative	Negative	Negative	RNA	Negative	Not performed	Negative	Negative	YES	NSCLC, Adenocarcinoma
16	Negative	RNA	Negative	Not performed	Negative	Negative	Negative	RNA	Negative	Not performed	Negative	Negative	YES	NSCLC, Squamous cell carcinoma
17	Negative	RNA	Negative	Not performed	Negative	Negative	Negative	RNA	Negative	Not performed	Negative	Negative	YES	NSCLC, Adenocarcinoma metastasis to rib
18	Negative	RNA	Negative	Not performed	Negative	Negative	Negative	RNA	Negative	Not performed	Negative	Negative	YES	NSCLC, Adenocarcinoma
19	Negative	RNA	Negative	Not performed	Negative	Negative	Equivocal	RNA	Negative	Negative	Negative	Negative	YES	NSCLC, Adenocarcinoma
20	Negative	RNA	Negative	Not performed	Negative	Negative	Negative	RNA	Negative	Not performed	Negative	Negative	YES	NSCLC, M/P Adenocarcinoma
21	Negative	RNA	Negative	Not performed	Negative	Negative	Negative	RNA	Negative	Not performed	Negative	Negative	YES	NSCLC, Adenocarcinoma
22	Negative	RNA	Negative	Not performed	Negative	Negative	Equivocal	RNA	Negative	Negative	Negative	Negative	YES	NSCLC, Adenocarcinoma
23	Negative	RNA	Negative	Not performed	Negative	Negative	Negative	RNA	Negative	Not performed	Negative	Negative	YES	NSCLC, Adenocarcinoma
24	Negative	RNA	Negative	Not performed	Negative	Negative	Negative	RNA	Negative	Not performed	Negative	Negative	YES	NSCLC, Adenocarcinoma
25	Negative	RNA	Negative	Not performed	Negative	Negative	Negative	RNA	Negative	Not performed	Negative	Negative	YES	NSCLC, Adenocarcinoma
26	Negative	RNA	Negative	Not performed	Negative	Negative	Negative	RNA	Negative	Not performed	Negative	Negative	YES	NSCLC, Adenocarcinoma
27	Negative	RNA	Negative	Not performed	Negative	Negative	Negative	RNA	Negative	Not performed	Negative	Negative	YES	Lung Adenocarcinoma metastasis to liver
28	Negative	RNA	Negative	Not performed	Negative	Negative	Negative	RNA	Negative	Not performed	Negative	Negative	YES	Metastatic Adenocarcinoma to Ilium, origin unknown
29	Negative	RNA	Negative	Not performed	Negative	Negative	Negative	RNA	Negative	Not performed	Negative	Negative	YES	NSCLC, Squamous cell carcinoma
30	Negative	RNA	Negative	Not performed	Negative	Negative	Negative	RNA	Negative	Not performed	Negative	Negative	YES	NSCLC, Adenocarcinoma
31	Negative	RNA	Negative	Not performed	Negative	Negative	Negative	RNA	Negative	Not performed	Negative	Negative	YES	NSCLC, Adenocarcinoma
32	Negative	RNA	Negative	Not performed	Negative	Negative	Negative	RNA	Negative	Not performed	Negative	Negative	YES	NSCLC, Squamous cell carcinoma
33	Negative	RNA	Negative	Not performed	Negative	Negative	Negative	RNA	Negative	Not performed	Negative	Negative	YES	NSCLC, Adenocarcinoma
34	Negative	RNA	Negative	Not performed	Negative	Negative	Negative	RNA	Negative	Not performed	Negative	Negative	YES	NSCLC, Adenocarcinoma
35	Negative	RNA	Negative	Not performed	Negative	Negative	Negative	RNA	Negative	Not performed	Negative	Negative	YES	NSCLC, Adenocarcinoma
36	Negative	RNA	Negative	Not performed	Negative	Negative	Negative	RNA	Negative	Not performed	Negative	Negative	YES	NSCLC, Squamous cell carcinoma
37	Not performed	RNA	Negative	Not performed	Negative	Negative	positive	RNA	positive	Not performed	positive	positive	YES	Lung adenocarcinoma metastasis to portal lymph nodes
38	Negative	RNA	Negative	Not performed	Negative	Negative	Negative	RNA	Negative	Not performed	Negative	Negative	YES	NSCLC, Squamous cell carcinoma
39	Negative	RNA	Negative	Not performed	Negative	Negative	Negative	RNA	Negative	Not performed	Negative	Negative	YES	NSCLC, Adenocarcinoma
40	Equivocal	NA	Not performed	positive	positive	positive	Negative	DNA	Not performed	Not performed	Negative	Negative	YES	NSCLC, Adenosquamous
41	Negative	NA	Not performed	Negative	Negative	Negative	Equivocal	DNA	Not performed	positive	positive	positive	YES	NSCLC, poorly dif. Adenocarcinoma
42	Not performed	NA	Not performed	positive	positive	positive	Negative	DNA	Not performed	Negative	Negative	Negative	YES	NSCLC, poorly dif. Adenocarcinoma
43	Not performed	NA	Not performed	Not performed	not tested	Negative	Negative	NA	Not performed	positive	positive	positive	YES	NSCLC, Adenocarcinoma
44	Not performed	NA	Not performed	Not performed	not tested	Negative	Not performed	NA	Not performed	positive	positive	positive	YES	NSCLC, Adenocarcinoma
45	Not performed	NA	Not performed	Not performed	not tested	Negative	Negative	DNA	Not performed	positive	positive	positive	YES	NSCLC, Adenocarcinoma
46	Not performed	RNA	Negative	Not performed	Negative	Negative	Not performed	RNA	positive	positive	positive	positive	YES	NSCLC, poorly dif. Adenocarcinoma
47	Negative	NA	Not performed	Not performed	Negative	Negative	Negative	DNA	Not performed	Not performed	Negative	Negative	YES	NSCLC, Adenocarcinoma
48	Negative	NA	Not performed	Not performed	Negative	Negative	Negative	DNA	Not performed	Not performed	Negative	Negative	YES	NSCLC, Mucinous Adenocarcinoma
49	Negative	NA	Not performed	Not performed	Negative	Negative	Negative	DNA	Not performed	Not performed	Negative	Negative	YES	NSCLC, Mucinous Adenocarcinoma
50	Negative	NA	Not performed	Not performed	Negative	Negative	Negative	DNA	Not performed	Not performed	Negative	Negative	YES	NSCLC, Adenocarcinoma

51	Negative	NA	Not performed	Not performed	Negative	Negative	Negative	DNA	Not performed	Not performed	Negative	Negative	YES	NSCLC, Adenocarcinoma
52	Negative	NA	Not performed	Not performed	Negative	Negative	Equivocal	DNA	Not performed	Negative	Negative	Negative	YES	NSCLC, Adenocarcinoma
53	Negative	NA	Not performed	Not performed	Negative	Negative	Negative	DNA	Not performed	Not performed	Negative	Negative	YES	NSCLC, Adenocarcinoma
54	Negative	NA	Not performed	Not performed	Negative	Negative	Negative	DNA	Not performed	Not performed	Negative	Negative	YES	NSCLC, Adenocarcinoma
55	Negative	NA	Not performed	Not performed	Negative	Negative	Negative	DNA	Not performed	Not performed	Negative	Negative	YES	NSCLC, Undifferentiated Carcinoma
56	Negative	NA	Not performed	Not performed	Negative	Negative	Negative	DNA	Not performed	Not performed	Negative	Negative	YES	NSCLC, Adenocarcinoma
57	Negative	RNA	Negative	Negative	Negative	positive	Negative	RNA	Negative	Not performed	Negative	Negative	NO	NSCLC, Adenocarcinoma
58	Negative	RNA	Negative	Not performed	Negative	Negative	Negative	RNA	Negative	Not performed	Negative	Negative	YES	NSCLC, Carcinoma with NE expression
59	Negative	RNA	Negative	Not performed	Negative	Negative	Negative	RNA	Negative	Not performed	Negative	Negative	YES	NSCLC, Adenocarcinoma
60	Equivocal	NA	FAILED	Negative	Negative	Negative	Negative	DNA	FAILED	Negative	Negative	Negative	YES	NSCLC, Adenocarcinoma
61	Negative	RNA	Negative	Not performed	Negative	Negative	Negative	RNA	Negative	Not performed	Negative	Negative	YES	NSCLC, Adenocarcinoma
62	Negative	RNA	Negative	Not performed	Negative	Negative	Negative	RNA	Negative	Not performed	Negative	Negative	YES	NSCLC, Adenocarcinoma
63	Negative	RNA	Negative	Not performed	Negative	Negative	Negative	RNA	Negative	Negative	Negative	Negative	YES	NSCLC, Adenocarcinoma with signet-ring like features
64	Negative	NA	FAILED	Negative	Negative	Negative	Negative	DNA	FAILED	Negative	Negative	Negative	YES	NSCLC, Adenocarcinoma
65	Negative	RNA	Negative	Not performed	Negative	Negative	Negative	RNA	Negative	Not performed	Negative	Negative	YES	NSCLC, Squamous carcinoma
66	Negative	RNA	Negative	Not performed	Negative	Negative	Negative	RNA	Negative	Not performed	Negative	Negative	YES	NSCLC, m/p Squamous carcinoma
67	Negative	RNA	Negative	Not performed	Negative	Negative	Negative	RNA	Negative	Not performed	Negative	Negative	YES	NSCLC, Squamous carcinoma
68	Negative	RNA	Negative	Not performed	Negative	Negative	Negative	RNA	Negative	Not performed	Negative	Negative	YES	NSCLC, Adenocarcinoma
69	Negative	RNA	Negative	Not performed	Negative	Negative	Negative	RNA	Negative	Not performed	Negative	Negative	YES	NSCLC, m/p Squamous carcinoma
70	Negative	RNA	Negative	Not performed	Negative	Negative	Negative	RNA	Negative	Not performed	Negative	Negative	YES	NSCLC, Adenocarcinoma
71	Negative	NA	FAILED	Not performed	Negative	Negative	Negative	DNA	FAILED	Not performed	Negative	Negative	YES	NSCLC, Adenocarcinoma
72	Negative	RNA	Negative	Not performed	Negative	Negative	Negative	RNA	FAILED	Not performed	Negative	Negative	YES	NSCLC, Adenocarcinoma

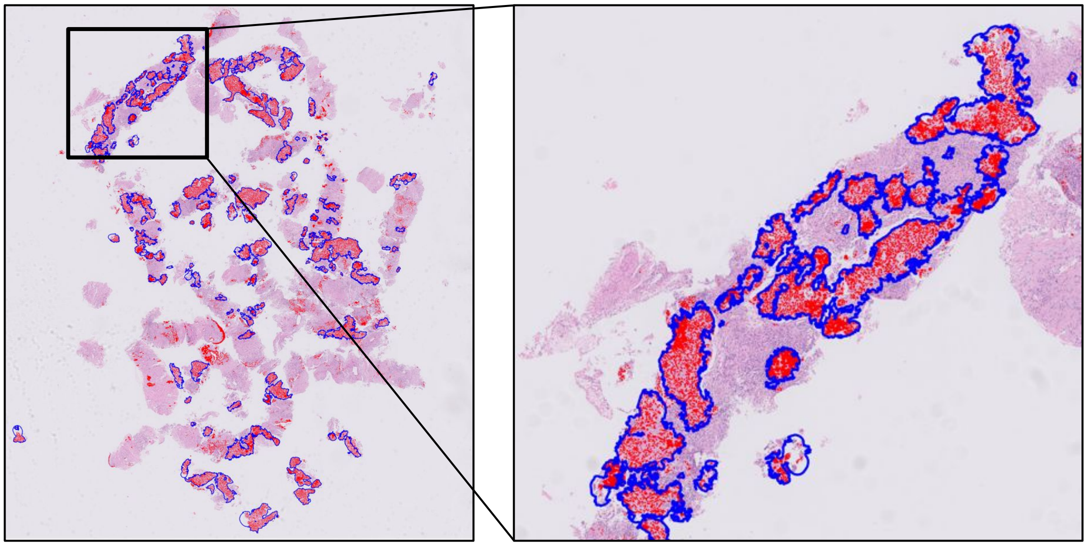

Supplementary Figure S1. **WSI showing agreement between manual annotations of cancerous regions and ROIs**

Representative H&E slide displaying ROIs in red and a pathologist's manual cancer regions annotation marked with blue line borders. High overlap can be seen between the two.

WSI - whole slide image ; ROI - region of interest

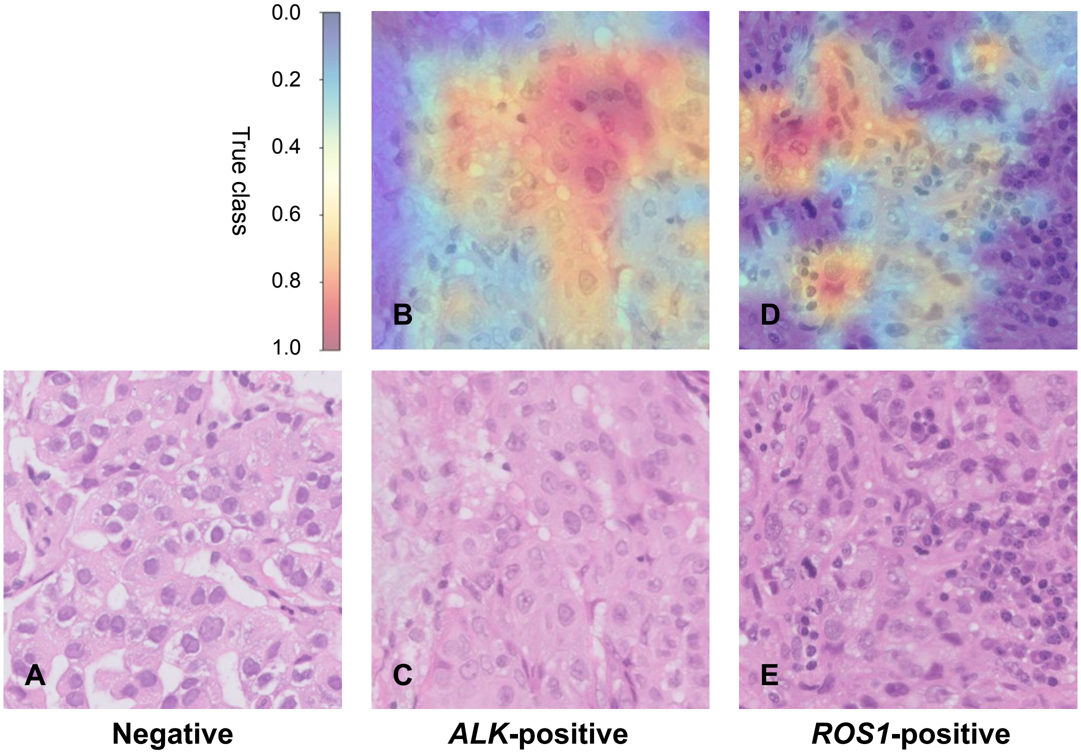

Supplementary Figure S2. *ALK* and *ROS1* heatmaps

Representative patches from the H&E images of a negative (A), *ALK*-positive (C) and *ROS1*-positive (E) cases, along with the matched heatmaps demarcating the regions contributed the most to the model prediction ((B) for *ALK*- and (D) for *ROS1*-positive). Size bar = 64µm.
